# Cortisol Detection Methods and the Hormone’s Role in Evaluating Circadian Rhythm Disruption

**DOI:** 10.3390/ijms26189141

**Published:** 2025-09-19

**Authors:** Norsham Juliana, Sofwatul Mokhtarah Maluin, Nadia Mohd Effendy, Izuddin Fahmy Abu, Sahar Azmani

**Affiliations:** 1 Department of Medical Science, Faculty of Medicine and Health Sciences, Universiti Sains Islam Malaysia (USIM), Nilai 71800, Negeri Sembilan, Malaysia; njuliana@usim.edu.my (N.J.); nadia@usim.edu.my (N.M.E.); drazmanisahar@kpju.edu.my (S.A.); 2 Institute of Medical Science Technology, Universiti Kuala Lumpur, Kajang 43000, Selangor, Malaysia; izuddin@unikl.edu.my; 3 Graduate School of Medicine, KPJ Healthcare University, Lot PT 17010 Persiaran Seriemas, Kota Seriemas, Nilai 71800, Negeri Sembilan, Malaysia

**Keywords:** cortisol, circadian rhythm, circadian disruption, biological markers, sleep–wake disorder

## Abstract

Cortisol follows a 24 h circadian rhythm that plays a pivotal role in maintaining the optimal function of various physiological systems in alignment with behavioural cycles. Its synthesis and secretion are regulated by the hypothalamic–pituitary–adrenal (HPA) axis. The 24 h fluctuations of cortisol may result from physiological changes influencing its regulation, or conversely, hormone-mediating physiological changes within the body. This review mainly aims to synthesize current evidence on methods for detecting cortisol. In addition, it focuses on evaluating cortisol’s potential as a biomarker for circadian disruption and related health impacts. A literature search was conducted across databases, including Google Scholar, PubMed, and Scopus, using search terms such as “circadian rhythm OR circadian clock OR circadian disruption OR circadian dysregulation” and “cortisol OR hydrocort* OR corticoid OR corticosteroid”. A total of 47 articles were included on methods of cortisol detection, and 41 articles were reviewed for their health implications. Cortisol measured via saliva, blood serum, urine, interstitial fluid (ISF), and sweat has been reported as suitable for 24 h monitoring, reflecting circadian regulation. In contrast, hair cortisol is suitable for identifying chronic changes and prolonged elevations in cortisol levels. This review highlights the stability, suitability, and challenges of each detection method, including reported cortisol levels across studies. Additionally, it provides a comprehensive overview of health implications associated with changes in cortisol, offering insights into its potential as a marker for circadian disruption and related health outcomes.

## 1. Introduction

Circadian rhythms (CRs) represent the intrinsic biological oscillations that regulate various physiological and behavioural processes within a given 24 h period. These rhythms orchestrate bodily functions, including sleep–wake cycles, hormone release, digestion, and body temperature relative to environmental changes [1]. The harmonious synchronization of these rhythms is associated with better health outcomes, while disruption correlates with various pathological states. The biggest influence on CRs has always been the exposure to environmental light and darkness. However, food intake, stress levels, physical activities, social environment, and temperature also play important parts that affect the regulation of CRs. The physiological study of the human body has pointed out that nearly every tissue and organ has its own CRs. Collectively, they are tuned to the daily day and night cycle [2]. Disruption of the CRs not only concerns the sleep and wake cycle but has been associated with severe health implications for multiple organ systems, including the immune, reproductive, gastrointestinal, skeletal, endocrine, renal, and cardiovascular systems. The suprachiasmatic nucleus (SCN), or the central clock, is not the only internal mechanism of control, and recent discoveries have revealed the presence of peripheral oscillators or secondary clocks throughout the body in a number of organs like the heart, liver, kidneys, lungs, intestines, skin, lymphocytes, esophagus, spleen, thymus, adrenal gland, prostate, and olfactory bulb. Although these organs function independently, their secondary clocks are synchronized with the SCN and other CRs factors such as temperature, meal timings, and external cues [3].

**Figure 1:**
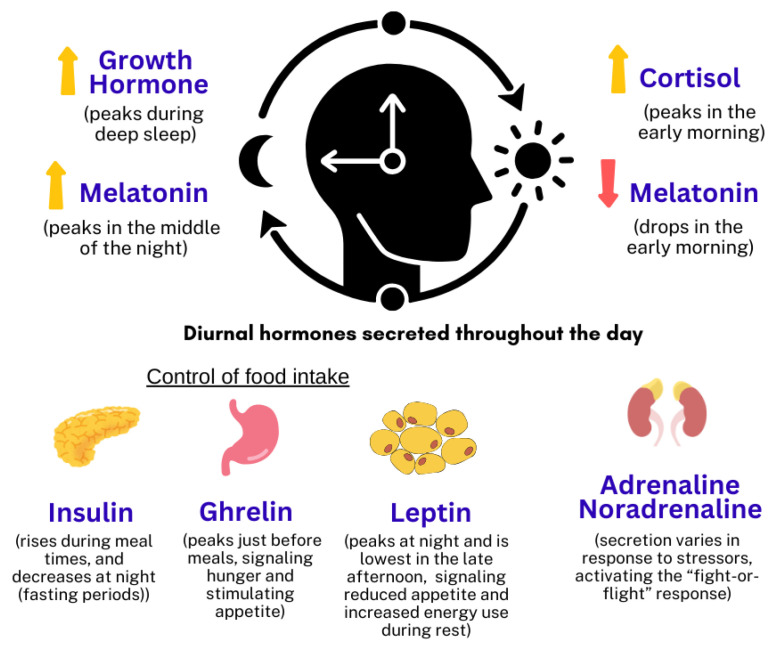
The collective hormones that contribute to the synchronization of physiological processes with the 24 h day–night cycle [9].

The regulation of CRs involves several key hormones, each playing distinct roles in maintaining the body’s internal clock. Above all, two CRs hormones are melatonin and cortisol (Figure 1 illustrates the hormones that have bidirectional effects on CRs). Traditionally, melatonin has been thought to be the superior CRs hormones that is also known as the “circadian rhythm hormone”. Melatonin is produced by the pineal gland, which receives information regarding the light–dark cycle from the surrounding environment [4]. Cortisol, the predominant glucocorticoid secreted by the adrenal gland, is referred to as the “activation hormone”. Cortisol helps regulate energy expenditure, metabolism, immune function, and alertness. It follows a circadian pattern, peaking in the early morning to increase alertness, and its decline promotes relaxation and sleep [5,6]. Melatonin and cortisol play complementary roles in regulating circadian rhythms. While both hormones are crucial for circadian rhythm regulation, in the current modern environmental influence, cortisol has a potentially superior role on CRs in terms of its broader impact on various physiological processes, together with its function in bodily response to stress and energy management. Melatonin, on the other hand, is more specifically focused on sleep regulation [7,8]. Besides these two hormones, growth hormone, insulin, leptin, ghrelin, adrenaline, and noradrenaline are also hormones influenced by circadian rhythm. These hormones collectively contribute to the synchronization of physiological processes with the 24 h day–night cycle, ensuring that various bodily functions occur at optimal times. Figure 1 illustrates the circadian changes in these hormones.

**Figure 2:**
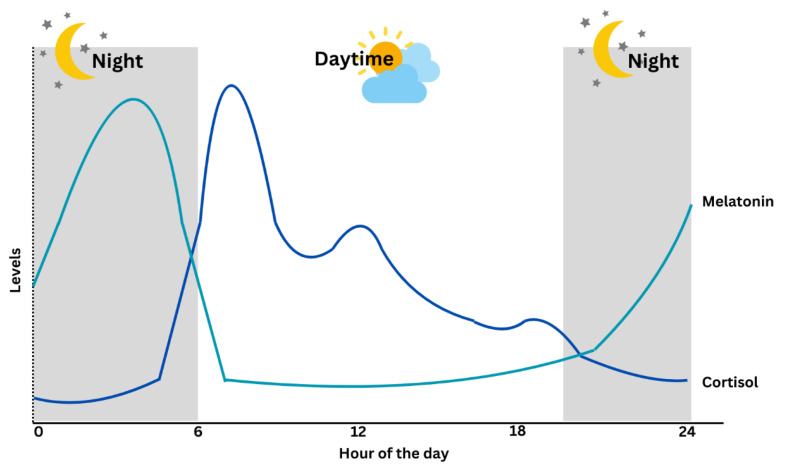
The physiological regulation of cortisol and melatonin levels within a 24 h [10,11].

Cortisol diurnal secretion, together with its ultradian oscillations that permit a rapid response to environmental threats, is controlled by the hypothalamic–pituitary–adrenal (HPA) axis. Cortisol regulations include predictable and unpredictable rhythms. The predictable, precise cortisol regulation is related to CRs, while the unpredictable rhythm involves superimposed pulsatile patterns that allow rapid responses to fine-tune physiological responses to changes in both the external and internal environment [6]. Circadian disruption or misalignment is characterized by the desynchronization between the central clock and human behavioural cycles involving 24 h sleep and wake, feeding and fasting, and activity and rest cycles [5]. Studying the CRs misalignment is complicated by various confounding factors, including external environmental or behavioural influences. Cortisol’s distinct secretory patterns, exhibiting both consistent diurnal rhythmicity and dynamic environmental influence, provide a comprehensive overview of the functioning of the human circadian system. Table 1 describes the comparison between cortisol and melatonin in circadian patterns, and Figure 2 illustrates the circadian circulation of both hormones.

Cortisol rhythm in Figure 2 is characterized by a gradual rise during the latter part of the sleep period, culminating in a peak within 30 to 45 min after awakening [19]. Beyond this surge, cortisol levels remain elevated through the early daytime hours, supporting sustained alertness, metabolic activity, and stress responsiveness. As the day progresses, the levels decline steadily and reach their nadir during the early sleep phase, facilitating rest and immune restoration [20]. Several studies reported a subtle secondary elevation in cortisol levels during the early to mid-afternoon period, typically between 2:00 and 4:00 pm. Meal-induced cortisol responses (especially after high-protein or high-glycemic meals) may contribute to this elevation. Throughout the day, ultradian pulses that lead to shorter cycles of hormone release can superimpose transient increases, especially in response to meal timing, cognitive load, or mild stressors [21]. Melatonin levels, on the other hand, begin rising approximately two hours before habitual sleep onset, peaking between 2 and 4 am, and decline in the morning. Light exposure, especially blue wavelengths (~460–480 nm), suppresses melatonin via the SCN-mediated inhibition of arylalkylamine N-acetyltransferase (AANAT) [22].

Cortisol’s distinctive characteristics make it a promising candidate for detecting and measuring circadian rhythm disruptions. Recognizing the gap in establishing a gold standard hormonal marker for assessing circadian disruption, this review addresses two key questions: (1) What are the current methods available for detecting cortisol in biological samples? (2) How can cortisol patterns be used to evaluate circadian rhythm disruption and related health outcomes? To answer these questions, we synthesize current evidence on cortisol detection methods and examine its potential as a biomarker for circadian regulation and health impacts. Particular focus is placed on studies assessing the diurnal secretion of cortisol over a 24 h period or those characterizing pulsatile hormone release at different time points of the day.

## 2. Methods

This review will discuss important findings concerning the sleep and circadian regulation of cortisol, particularly high-quality studies where diurnal secretion of cortisol was assessed across an entire 24 h period, or where hormone pulsatility could be accurately assessed because blood sampling was sufficiently frequent. Recently published findings will be emphasized. A comprehensive search was conducted across three electronic databases, including Scopus, Web of Science, and PubMed.

The following are examples of search keywords used to retrieve studies in Scopus:

1. (TITLE-ABS-KEY (circadian AND (rhythm OR clock OR disruption OR dysregulation)));
2. (TITLE-ABS-KEY (cortisol OR hydrocort* OR *corticoid OR corticosteroid));
3. #1 AND #2.

The following are examples of search keywords used to retrieve studies in Web of Science:

1. circadian rhythm OR circadian clock OR circadian disruption OR circadian dysregulation (All Fields);
2. cortisol or hydrocort* or corticoid or corticosteroid (All Fields);
3. #1 AND #2.

The following are examples of search keywords used to retrieve studies in PubMed:

1. circadian rhythm OR circadian clock OR circadian disruption OR circadian dysregulation);
2. cortisol OR hydrocort* OR *corticoid OR corticosteroid;
3. #1 AND #2.

This review includes 47 articles discussing various methods of cortisol detection and 41 articles exploring the health effects of disrupted cortisol rhythms. Articles were included if they were peer-reviewed, written in English, and provided primary or secondary data relevant to cortisol measurement or circadian health. Exclusion criteria included non-peer-reviewed sources, conference abstracts without full papers, and studies lacking relevant cortisol outcomes.

## 3. Methods of Cortisol Level Detection

The ability to detect elevated cortisol levels accurately and promptly is crucial for improving the diagnosis, management, and prevention of stress-associated conditions, including anxiety disorders, metabolic dysfunction, and cardiovascular diseases. However, most cortisol assessments at present are conducted in laboratory settings, with no point-of-care (POC) approaches available for real-time cortisol monitoring [23]. In clinical practice, the total cortisol level (comprising both free and protein-bound cortisols) is typically measured. However, only the free cortisol fraction is biologically active and responsible for physiological effects associated with cortisol in the biofluids [24]. Despite this, conventional laboratory tests offer only a suboptimal level of cortisol at the time of sampling, failing to capture its dynamic fluctuations influenced by the time of day. Due to this, 24 h urine analysis of cortisol is often preferred over single-point sampling [23].

### 3.1. Cortisol Level Detection in Biofluids

Traditional methods for cortisol detection include colorimetric fluorescence-based assays, high-performance liquid chromatography (HPLC), inverse-phase chromatography, enzyme-linked immunosorbent assays (ELISA), competitive protein-binding assays, and biosensors utilizing surface plasmon resonance (SPR) assays [25]. However, these approaches are often hindered by lengthy measurement processes, limited sensitivity, and high expenses [25]. Recent technological advancements show a promising development of systems for more comprehensive cortisol monitoring [23].

#### 3.1.1. Saliva

The measurement of cortisol level in the saliva has emerged as a popular and convenient biomarker [25]. Compared to venipuncture, salivary testing provides several advantages, including being non-invasive, minimizing the likelihood of confounding factors, and allowing the collection of multiple samples without raising ethical issues [26,27]. Studies have demonstrated a correlation between salivary and serum cortisol levels [28], enabling the measurement of active cortisol in the saliva through a stress-free and non-invasive process [29]. Notably, in contrast to the blood, where 90% of cortisol is protein-bound, salivary cortisol exists entirely in its free form [25]. The simple sampling and handling process and storage of saliva make it an excellent medium for cortisol detection with less analytical variability [27]. However, the limitation with salivary cortisol is that it is present only in its free form; hence, the total cortisol concentration is significantly lower than that in blood, necessitating sensitive detection methods with lower limits of detection (LOD) to ensure accurate measurements [25]. Additionally, contamination from blood in the saliva caused by oral lesions may affect cortisol levels and result in quantification inaccuracies [25].

Radioimmunoassays (RIAs) were widely used as the gold standard for determining salivary cortisol levels due to their simplicity, rapidness, cost-effectiveness, sensitivity, reliability, and reproducibility [30]. However, advancements in immunoassays (IA) led to the development of methods without the use of radioactive materials, shifting the focus toward fluorescence labelling techniques. Currently, enzyme-linked immunosorbent assays (ELISAs) and immune-based biosensors are gaining attention as the preferred choice [29]. The main advantages of IA include their practicality for clinical use, where various commercial kits are readily available in the markets, providing ease of use, with time- and cost-effectiveness. Nevertheless, they are not without limitations. Cross-reactivity between the antibodies with other structurally similar substances may occur [29,31].

Currently, cortisol measurement is frequently conducted using LC–MS/MS, which offers high selectivity and sensitivity. Other chromatography techniques include high-performance thin-layer chromatography (HPTLC) and eXtraction Liquid Chromatography (XLC). Surface plasmon resonance (SPR) technique, which measures changes in the refractive index (RI), provides a quick protocol for detecting salivary cortisol without requiring extensive sample preparation [29]. Advancements in SPR sensors utilizing fiber optics have recorded the lowest LOD of cortisol [32]. Moreover, electrochemical (EC) detection methods are gaining prominence due to their simplicity, speed, and reduced solvent usage, making them well-suited for point-of-care (POC) applications [29].

#### 3.1.2. Blood or Serum

Blood testing was one of the earliest methods used to measure cortisol levels in biofluids [25]. Cortisol levels in blood exhibit a diurnal pattern; hence, its natural fluctuations throughout the day necessitate careful consideration of timing when interpreting results [5]. The total serum cortisol reference ranges from 3 to 23 µg/dL at 8 AM, and 3 to 13 µg/dL at 4 PM [33]. Blood-based sampling has several limitations, making it a less preferred method for cortisol analysis. Blood collection is subject to potential infections, hence it requires medical expertise and sterile tools [25]. Due to cortisol’s instability at room temperature and increased temperature, specific handling and storage of blood samples are essential [23]. Furthermore, the invasive nature of blood puncture sampling often induces pain and anxiety in patients, potentially elevating cortisol levels before and during the blood withdrawal process [23].

#### 3.1.3. Hair

Cortisol can be found within the hair shaft, with the highest concentrations typically detected in the segment closest to the scalp, representing cortisol production over the previous month [25,34]. It is postulated that hair cortisol demonstrates the free cortisol level rather than the total cortisol found in blood plasma or serum. Reference values for hair cortisol range from 40 to 128 pg/mL [35]. Hair sampling for cortisol analysis is convenient as it offers a non-invasive approach [23]. Earlier on, Koren et al. [36] demonstrated the viability of detecting cortisol in wild hyrax hair using a modified ELISA protocol. Gao et al. [37] then developed a technique to detect cortisol in human hair using high-performance liquid chromatography with fluorescence detection (HPLC-FLU), which demonstrated specificity comparable to mass spectrometry. Most recently, Gonzalez et al. [35] developed a hair cortisol detection method using a chemiluminiscent immunoassay analyzer, the first automated protocol being established.

#### 3.1.4. Urine

Cortisol is present in the urine in both free (unconjugated) and conjugated forms [38]. Urinary cortisol levels, often referred to as 24 h urinary free cortisol (UFC), reflect the free and biologically active cortisol excreted in the urine [23,25]. The typical range for UFC levels is between 36 µg/24 h and 137 µg/24 h [39]. While collecting urine over 24 h is non-invasive and pain-free, it presents challenges related to feasibility and reliability, with regard to urine sample collection, storage, and transportation [25]. These logistical demands make UFC analysis unsuitable for real-time cortisol monitoring, especially in POC settings [23]. Additionally, other factors such as pregnancy [40] and medications [41,42] can significantly affect cortisol levels in urine samples, further complicating interpretation.

#### 3.1.5. Interstitial Fluid (ISF)

Cortisol is present in the interstitial fluid (ISF) in proportions similar to those in blood plasma [23]. Microneedles have been effectively developed and used for the painless and minimally invasive extraction of ISF [43]. However, the stratum corneum, a keratinized and low-permeability layer of the epidermis, can hinder ISF permeation through the skin [25]. For cortisol measurement, ISF must be withdrawn at a very slow rate of 10 µL/h, which poses challenges for POC or ambulatory applications. Several issues which limit the use of ISF for cortisol measurement include the biodegradability and biocompatibility of microneedles, infection risks, sterilization requirements, and continuous withdrawal of biofluid [23,25].

#### 3.1.6. Sweat

Sweat is emerging as a valuable non-invasive biofluid for health diagnostics, offering significant insights into physiological conditions [25,44]. Compared to urine, blood, and saliva, sweat is relatively easier to stimulate, harvest, and quantify [25]. The reference range for cortisol concentration in sweat is reported to be between 8 ng/mL and 142 ng/mL [45]. Sweat collection is often performed using sweat patches, which provide a non-invasive and efficient method. However, challenges remain in developing a reliable and reproducible sweat sampling device for cortisol detection due to limited understanding of the correlation of cortisol in sweat, and the influence of factors such as temperature, humidity, location, physical activity, and genetic variations [23]. To address these challenges, a novel aptamer-based lateral flow strip assay that uses cortisol-selective aptamers conjugated to gold nanoparticles (AuNPs) has been developed for rapid on-site cortisol detection in sweat. The handheld biosensor demonstrated several advantages, including a visual LOD of 1 ng/mL, no significant cross-reactivity with other biomarkers, and lower cost due to the use of aptamers instead of antibodies. Most importantly, it is stable, sensitive, simple, rapid, and user-friendly for a POC application for cortisol measurement in sweat [46].

Table 2 summarizes the various methods of sample collection for cortisol measurements, including their advantages and disadvantages.

### 3.2. Emerging Technologies for Cortisol Detection

Current methods for cortisol measurement in the clinical setting are costly and require skilled personnel, and often require the collection of urine or saliva samples, which must then be transported and analyzed in specialized laboratories [47]. Conventional sampling, such as urine and blood, is invasive, time-consuming, and unable to facilitate continuous monitoring [25]. Routine assays also frequently display significant biases, making it difficult to achieve robust standardization in clinical practice. To overcome these challenges, structure-based assays, such as mass spectrometry, are increasingly being recommended [47]. More advanced techniques for cortisol detection employ a range of transducers, including optical and electrochemical methods. Optical detection offers high sensitivity but relies on complex laboratory set-up and costly materials, limiting its suitability for POC applications [48].

Recent advancements in electrochemical sensors and material engineering have been reviewed by Sekar et al. (2020) [25], emphasizing wearable POC systems that enable early and rapid cortisol detection. Electrochemical sensing technologies incorporate receptor molecules such as antibodies, enzyme fragments, molecularly imprinted polymers, and biomimetic materials, providing high sensitivity with low LOD [25,47]. The simple, wearable, and portable biosensors are gaining attention as viable alternatives to invasive and costly laboratory procedures [49,50,51]. Paper-based wearable sensors have become particularly appealing due to their flexibility, increased surface area, widespread availability, and ease of production [52]. Meanwhile, Apilux et al. [53] have introduced a paper-based immunosensor with a competitive assay for a quick and easy protocol for cortisol detection in serum. This system utilizes a colorimetric method based on antibody-conjugated gold nanoparticles (AuNPs) and has demonstrated reliable recovery and precision for serum cortisol measurement.

Across emerging cortisol detection technologies, most evidence is based on small, often healthy-only samples, with limited real-world validation and inconsistent benchmarking against gold-standard methods [25,47]. Common issues include a lack of standardized performance metrics, variable calibration protocols, and minimal long-term stability testing, which together limit immediate clinical applicability. Comparative analysis of the strengths and limitations of emerging cortisol-detection technologies is presented in Table 3.

These methodological limitations also influence the readiness of different platforms for clinical and consumer integration. Wearable electrochemical sensors using sweat or interstitial fluid show the highest consumer potential due to their non-invasive design and mobile compatibility. However, clinical use is limited by sweat variability, calibration drift, and environmental factors [54,55]. Nanomaterial-based sensors offer high analytical sensitivity but remain preclinical for cortisol due to reproducibility issues, signal interference, and fabrication complexity [56,57]. Paper-based competitive immunosensors are moderately suited for supervised point-of-care use but less viable for unsupervised consumer applications due to operator dependence and stability concerns [58]. Common barriers include limited large-scale validation, lack of standardized metrics, regulatory uncertainty, and poor system interoperability.

**Table 2:** Methods of cortisol sampling, including their advantages and disadvantages.

Sample Collection	Typical Cortisol Range	Advantages	Disadvantages	Method	Suitability for Assessing Circadian Health
Saliva	7 AM–9 AM: 100–750 ng/dL 3 PM–5 PM: <401 ng/dL 11 PM–midnight: <100 ng/dL [33]	Concentration is not affected by flow rate from salivary glands [59]. Stress-free and non-invasive [29]. Less analytical variation due to logistical feasibility [23].	Contains only in its free form [25]. Contamination risks by oral lesions [25]. Low concentration of cortisol present [59].	ELISA ECL SPR MIPs Wearable	Suitable
Blood Serum	8 AM: 3–23 µg/dL 4 PM: 3–13 µg/dL [33]	Measures total cortisol [60]. High sensitivity and specificity due to well-established laboratory protocols [60].	Subject to infections [25]. Time and labour intensive, prefiltration steps often required [59]. Cortisol level fluctuates throughout the day and is unstable at room and increased temperatures [23]. Stress-induced cortisol elevation due to venipuncture [23].	RIA ELISA CLIA LC-MS/MS Optical sensors	Suitable
Hair	40–128 pg/mL [35]	Non-invasive [23]. Represent cortisol production over the previous month [25].	Short-term data [25].	LC-MS/MS ELISA	Not suitable
Urine	Adult/elderly: <100 µg/24 hr Adolescent: 5–55 µg/24 hr Child: 2–27 µg/24 hr [33]	Non-invasive [25]. Suitable for assessing adrenal function, such as in Cushing’s syndrome [60].	Storage and transportation demands, contamination risks [25]. Pregnancy and medications can affect cortisol levels [40].	RIA ELISA CLIA LC-MS/MS	Suitable
Interstitial Fluid (ISF)	1–11 ng/mL [59]	Painless and minimally invasive extraction using microneedles [43]. Can be collected during sleep [59]. Continuous sampling can be performed via microdialysis [61].	Epidermis layer can hinder ISF permeation through the skin [25]. Infection risks, sterilization requirements [23].	EIS Epidermal Wearable Biosensors [62] Vacuum-Assisted Extraction [63] Bifidobacteria-Modified Microelectrodes	Suitable
Sweat	8 ng/mL–142 ng/mL [45]	Non-invasive [23]. Easy to stimulate, harvest, and quantify [25]. Real-time monitoring potential [46]	Influenced by temperature, humidity, location, physical activity, and genetic variations [23]	Wearable Biosensors Polymer-Based Sensors Enzyme mimic sensors Optical sensors	Suitable

Enzyme-Linked Immunosorbent Assay (ELISA), Electrochemiluminescence (ECL), Surface Plasmon Resonance (SPR), Molecular Imprinted Polymers (MIPs), Radioimmunoassay (RIA), Chemiluminescent Immunoassay (CLIA), Liquid Chromatography–Tandem Mass Spectrometry (LC-MS/MS), Electrochemical Impedance Spectroscopy (EIS), Wearable Sensors.

**Table 3:** Strengths and limitations of emerging technologies for cortisol detection.

Emerging Technology	Sample Size and Population	Methodological Strengths	Limitations/Potential Biases	Stage of Validation
Wearable electrochemical sensors (sweat/ISF) [25]	Small pilot/prototype studies; healthy volunteers only	Non-invasive, continuous monitoring potential; wearable integration	No commercial devices; inconsistent benchmarking; sweat matrix effects; calibration and mechanical noise issues	Pre-commercial, prototype stage
Nanomaterial-based electrochemical sensors [47]	Review of lab-based nanostructured sensors; no study-level sample	Highlights high sensitivity/selectivity via MIPs, aptamers, etc.	Lack of standardized metrics; sparse real-world benchmarking or LC-MS/MS validation; variable calibration protocols	Lab-based validation (prototype)
Paper-based competitive immunosensor [62]	~3 serum samples (triplicates), healthy adults only	High spike-recovery; simple, cost-effective; rapid detection; good ECL agreement	Very small, homogeneous sample; unblinded; no long-term stability/drift data	Early analytical validation

## 4. The Potential of Cortisol in Evaluating Circadian Disruption and Related Health Outcomes

Assessment of circadian health is essential as its disruption can impact health, sleep, and well-being. Dim light melatonin onset (DLMO) is the gold standard for assessing circadian health in humans [64,65]. This phase assessment technique requires the collection of multiple hours of blood or saliva samples under low light conditions, usually in an inpatient setting, for a melatonin assay later [65]. In addition to DLMO, other melatonin markers include melatonin offset and the midpoint of melatonin production [64]. Urine 6-sulfatoxymelatonin is a melatonin metabolite that can also be measured in urine samples to assess circadian amplitude. It can be particularly useful in pediatric patients and in treating neurologic diseases [66]. Actigraphy is an objective measure that can be used to assess sleep/wake patterns over multiple days. Rest-activity cycles derived from actigraphy are a commonly used measure of circadian rhythms [67,68]. Sleep logs and sleep diaries are also useful as part of the diagnostic process of circadian health [68]. Standardized questionnaires such as the Morningness–Eveningness Questionnaire and the Munich Chronotype Questionnaire can also be used to assess chronotype [68,69]. Monitoring core body temperature can also help determine circadian health [69]. More recently, studies have implemented wearable devices to continuously monitor daily behaviours and physiological signals to assess circadian patterns [70].

Cortisol, the main focus of this review, is gaining attention as a biomarker to assess circadian health, as its levels follow the circadian rhythm [71]. A normal cortisol rhythm is characterized by a pronounced diurnal pattern, with levels rising during the latter part of the sleep period, peaking within 30–45 min after awakening, and gradually declining throughout the day to reach a nadir during early sleep, thereby supporting alertness, metabolism, and immune restoration [19,20]. Circadian disruption alters this profile, manifesting as a flattened diurnal slope, phase shifts in peak secretion, elevated evening cortisol, or a blunted cortisol awakening response (CAR) [5]. Such dysregulation is linked to increased cardiometabolic risk through mechanisms like insulin resistance, central obesity, and hypertension [72,73,74], impaired immune regulation leading to chronic inflammation or immune suppression [75,76], and neurological consequences including cognitive decline and heightened prevalence of mood disorders such as depression and anxiety [77,78]. Additionally, altered cortisol rhythms can disrupt gastrointestinal function by influencing gut microbiota composition and promoting inflammation [79], further underscoring the hormone’s pivotal role in linking circadian misalignment to systemic disease.

A stable cortisol rhythm is a critical regulator of the stress response and is essential for maintaining physiological homeostasis. Disruption of this rhythm adversely affects multiple organ systems. In the endocrine system, dysregulation of the hypothalamic–pituitary–adrenal (HPA) axis leads to abnormal hormone secretion. Metabolically, it increases the risk of insulin resistance, obesity, and type 2 diabetes. Cardiovascular implications include chronic inflammation and sustained hypertension, which elevate the risk of cardiovascular disease [80,81,82]. Immune function is also compromised, increasing susceptibility to infections and autoimmune disorders. Neurologically, aberrant cortisol patterns are linked to cognitive decline and a higher prevalence of mood disorders such as anxiety and depression [79]. In the gastrointestinal system, cortisol dysregulation alters the gut microbiota, contributing to conditions like irritable bowel syndrome (IBS) [83,84]. Sleep disturbances, particularly insomnia and fragmented sleep, commonly co-occur with disrupted cortisol rhythms, exacerbating systemic stress.

In addition, women generally exhibit higher cortisol responses to psychosocial stressors than men, partly due to hormonal influences such as estrogen and progesterone, which modulate hypothalamic–pituitary–adrenal (HPA) axis activity and cortisol reactivity [85,86]. Aging is associated with a blunted diurnal cortisol slope, reduced amplitude, and elevated evening cortisol levels, which may contribute to increased risks of metabolic syndrome, cognitive decline, and cardiovascular disorders in older adults [87]. Sex- and age-related variations further influence the health implications of cortisol dysregulation. For example, premenopausal women tend to have lower daytime cortisol levels and greater rhythm irregularity compared to men, while postmenopausal differences diminish [86]. In older individuals, cortisol peaks earlier and remains elevated longer, potentially exacerbating sleep disturbances and insulin resistance [87]. These physiological differences underscore the importance of stratifying cortisol-based assessments and tailoring interventions by sex and age in clinical practice.

A summary of the pathological impacts across metabolic, cardiovascular, immune, neurological, and reproductive domains is provided in Table 4.

## 5. Conclusions and Gaps of Knowledge

Cortisol is a compelling candidate to be a biomarker in detecting early signs of circadian disruption. Early detection of circadian disruption that is sub-pathological may promise a reversible state to preserve health. However, identifying the optimal range of cortisol levels that reliably reflects circadian dysregulation remains a significant gap, especially when accounting for age and gender differences. Additionally, the timing of sample collection, a critical factor for accurately interpreting cortisol rhythms, is yet to be standardized. These gaps underscore the need for robust research to establish evidence-based guidelines that would allow cortisol to serve as a precise biomarker for circadian health.

Based on current best practices, optimal time points for salivary or serum cortisol collection to assess diurnal rhythm include immediately upon awakening, approximately 30 min post-awakening to capture the cortisol awakening response (CAR), mid-afternoon (around 15:00 h) to evaluate the daytime decline, and late evening (around 22:00–23:00 h) to measure the nadir [88,89]. These intervals are widely recognized for their utility in characterizing circadian patterns and overall cortisol regulation.

While the potential for cortisol to become the gold standard marker of circadian health is promising, several challenges must be addressed. Key among these is the selection of the most appropriate sampling method of either saliva, blood, urine, or emerging techniques such as ISF or sweat collection. Ideally, the chosen method should be sensitive, accurate, minimally invasive, and cost-effective. Current approaches require multiple samples collected throughout the day to capture the dynamic nature of cortisol rhythms, but advancements in rapid, non-invasive testing methods may offer a solution. Future research should prioritize optimizing these methods to facilitate timely and practical assessment of circadian health, thereby enabling broader clinical and public health applications.

## Figures and Tables

**Table 1:** Comparison of cortisol and melatonin circadian patterns and their influencing factors.

Factor	Cortisol	Melatonin
Circadian Pattern	Peaks in the early morning (around 7–8 AM), declines throughout the day [10].	Rises in the evening, peaks during the night, decreases in the early morning [11].
Stability [12,13]	Highly stable and reproducible over time.	More sensitive to environmental factors like light exposure.
Influencing Factors	Stress, sleep quality, physical activity [14,15,16].	Light exposure, age [17,18].

**Table 4:** Effects of cortisol rhythm disruption on body systems.

System	Specific Effect	Changes to Cortisol	Type of Study	References
Endocrine	Insulin resistance, impaired glucose tolerance, central fat deposition → obesity and metabolic syndrome.	Elevated or flattened cortisol rhythm	Animal models, Human studies (Shift workers)	[72,73,74]
Nervous	i. Impaired cognitive functions: Hippocampal atrophy, reduced neurogenesis → accelerating dementia and cognitive decline.	Chronic cortisol elevation	Human study	[79]
	ii. Increased risk of mood disorders Linked to MDD, anxiety, bipolar disorder, PTSD, ADHD, schizophrenia, and Alzheimer’s.	Elevated cortisol, except for PTSD which is due to reduced cortisol level	Animal models, Human studies, Meta-analysis	[77,78]
Cardiovascular	Hypertension, hyperlipidemia, and endothelial dysfunction leading to atherosclerosis → higher risk for CVD.	Elevated cortisol	Human studies (General population, Shift workers)	[80,81,82]
Digestive	Altered gut microbiota, increased gastrointestinal inflammation.	Fluctuations in cortisol	Human study	[83]
Immune	Immune suppression, reduced cytokine production, and chronic inflammation.	Fluctuations in cortisol levels with circadian rhythm	Animal models, Human studies	[75,76]

## Data Availability

No new data were created or analyzed in this study.
